# Thermal-stress analysis of a damaged solid sphere using hyperbolic two-temperature generalized thermoelasticity theory

**DOI:** 10.1038/s41598-021-82127-1

**Published:** 2021-01-27

**Authors:** Hamdy M. Youssef, Alaa A. El-Bary, Eman A. N. Al-Lehaibi

**Affiliations:** 1 Mathematics Department, Faculty of Education, Alexandria University, Alexandria, Egypt; 2 Mechanical Engineering Department, College of Engineering and Islamic Architecture, Umm Al-Qura University, Makkah, Kingdom of Saudi Arabia; 3 Basic and Applied Science Institute, Arab Academy for Science, Technology, and Maritime Transport, P.O. Box 1029, Alexandria, Egypt; 4 Mathematics Department, Al-Lith University College, Umm Al-Qura University, Al-Lith, Saudi Arabia

**Keywords:** Mechanical engineering, Engineering, Materials science, Mathematics and computing

## Abstract

This work aims to study the influence of the rotation on a thermoelastic solid sphere in the context of the hyperbolic two-temperature generalized thermoelasticity theory based on the mechanical damage consideration. Therefore, a mathematical model of thermoelastic, homogenous, and isotropic solid sphere with a rotation based on the mechanical damage definition has been constructed. The governing equations have been written in the context of hyperbolic two-temperature generalized thermoelasticity theory. The bounding surface of the sphere is thermally shocked and without volumetric deformation. The singularities of the studied functions at the center of the sphere have been deleted using L’Hopital’s rule. The numerical results have been represented graphically with various mechanical damage values, two-temperature parameters, and rotation parameter values. The two-temperature parameter has significant effects on all the studied functions. Damage and rotation have a major impact on deformation, displacement, stress, and stress–strain energy, while their effects on conductive and dynamical temperature rise are minimal. The thermal and mechanical waves propagate with finite speeds on the thermoelastic body in the hyperbolic two-temperature theory and the one-temperature theory (Lord-Shulman model).

## Introduction

In material science, researchers and authors play a key role in seeking a precise and effective model simulating the behavior of the thermoelastic materials. Authors and researchers have provided many mathematical models in which they studied the transmission of thermomechanical waves in solid materials. It requires a large space not limited to one research to talk about thermomechanical transition mathematical models by using elastic materials. So, we will speak about some recent models which need chances to discuss. Chen and Gurtin^1^ introduced a thermoelasticity model based on two different types of temperatures; the dynamical temperature and conductive temperature. The difference value between these two temperatures is proportional to the value of the heat supply. Warren and Chen^2^ studied the wave propagation in the context of the two-temperature thermoelasticity theory. Youssef^3^ modified this theory and introduced the model of two-temperature generalized thermoelasticity. Youssef with other researchers have used that model in many applications and researches^4–6^. Youssef and El-Bary^7^ introduced the evidence of the two-temperature generalized thermoelasticity model does not provide a finite speed of propagating the thermal waves. Therefore, Youssef and El-Bary^7^ modified this model and introduced a new model of two-temperature based on different heat conduction laws called hyperbolic two-temperature generalized thermoelasticity. In that model, Youssef proposed that the value of the difference between the value of the conductive temperature acceleration and the value of the dynamical temperature acceleration is proportional to the heat supply. Within this model, the thermal wave propagates through the medium with a finite speed. Le Thanh et al.^8^ developed a thermomechanical size-dependent model using finite element method for predicting the stress, thermal defection, and critical buckling load of composite microplates laminates based on the Reddy plate theory together with new modification of couple stress theory. For the first time, Le Thanh et al.^9^ used isogeometric analysis for the size-dependent impacts on the post-buckling and thermal buckling behaviors of functionally graded micro-plates with porosities.

Youssef introduced many applications of thermoelasticity of infinite thermoelastic spherical medium^10,11^. Mukhopadhyay and Kumar studied and discussed the generale form of the thermoelastic interactions in an ifinite thermoelastic body with a spherical cavity^12^. Many researheres studied the influence of the rotation on the thermal and mechanical waves. In the two-dimensional applications of generalized thermoelastic materials, Baksi et al.^13^ used the eigenvalue method for investigating the influence of the relaxation time and rotation. Othman^14^ studied the rotation's effect on the plane waves in the context of the generalized thermoelasticity model based on two relaxation times. Baksi et al.^15^ studied the influence of the rotation and relaxation time in the generalized magneto-thermo-viscoelastic in a one-dimensional medium. Othman and Singh^16^ studied the effects of the rotation on a generalized thermoelastic micropolar half-space under five different theories. Singh and Singla^17^ discussed the effects of the rotation on the propagating waves in an incompressible transversely thermoelastic isotropic solid material.

Many researchers who found their applications and problems as a spherical medium believed that a body with a spherical cavity is away from the situation at the sphere's center. Few authors could overcome the problem; Thibault et al., for example, employed the L'Hopital rule in the thermoelectric solid sphere to address the singularity situation^18^.

The aim of this investigation is to study the influneces of the rotation and mechanical damage on the thermoelastic solid sphere under a new theory of thermoelasticity which is called hyperbolic two-temperature generalized thermoelasticity theory. The main goal of this work is to prove that the hyperbolic two-temperature thermoelasticity theory grantees a thermoelastic wave which propagates with a finite speed and it is a successful model.

## The governing equations

**Figure 1:**
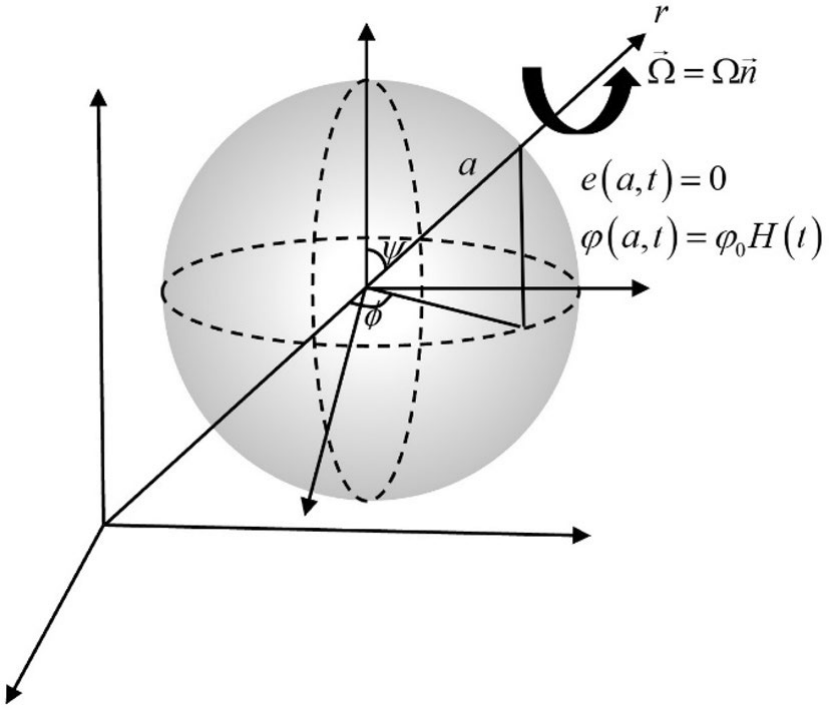
The thermoelastic solid sphere with rotation.

Consider a perfect thermal thermoelastic, isotropic, and spherical body that fills the region $\Lambda = \left\{ {\left( {r,\psi ,\phi } \right):\,\,\,0 \le r \le a,\,\,\,0 \le \psi \le 2\pi ,\,\,\,\,0 \le \phi < 2\pi \,\,} \right\}$. We can apply the well-known spherical co-ordinates system $\left( {r,\psi ,\phi } \right)$ where $r$ denote the radial co-ordinate $\psi$ and $\phi$ denoted to the co-latitude, and longitude of a spherical coordinate system, respectively. Assume the medium has no body force and initially quiescent. Consider the sphere is rotating uniformly with an angular velocity $\vec{\Omega } = \Omega \,\vec{n}$ , where $\vec{n}$ is a unit vector representing the direction of the axis of rotation, as in Fig. 1.

There are two more terms for the motion equation of the revolving frame^19^; $\Omega \times \left( {\Omega \times u} \right)$ gives the centripetal acceleration comes from the time-varying motion only, and $\left( {2\Omega \times \dot{u}} \right)$ which is called the Coriolis acceleration. Only if the latitudinal and longitudinal variance is available is the symmetry requirement fulfilled. Thus, all the studied functions will depend on radial distance $r$ and time $t$.

The damage amount can be measured by the fractional area^20^:

1 $$ D = \frac{{dA_{D} }}{dA},\quad 0 \le D \le 1, $$

where $D = 0$ is devoted to the undamaged case while $D = 1$ describes the fully damaged case formally with a total loss of stress carrying capacity. Physically in natural materials, the value of the damage parameter will be $D \approx 0.2...0.5$. In the case of isotropic damage, the effective stresses are given by^20^:

2 $$ \sigma_{ij} = \left( {1 - D} \right)\tilde{\sigma }_{ij} , $$

where $\tilde{\sigma }_{ij}$ are the stresses components in the undamaged material. Many articles have been published under this definition of damage mechanics^21–25^. Khatir et al.^26^ presented an enhanced application for damage quantification in laminated composite with less computational time based on IGA using modal analysis. Zenzen et al.^27^ introduced a new damage indicator are by using two numerical models.

Due to spherical symmetry, the displacement components have the form

3 $$ \left( {u_{r} ,u_{\psi } ,u_{\phi } } \right) = \left( {u\left( {r,t} \right),0,0\,} \right). $$

The equations of motion^14,16,28^:

4 $$ \rho \left( {\ddot{u} - \Omega^{2} u} \right) = \left( {\lambda + 2\mu } \right)\left( {1 - D} \right)\frac{\partial e}{{\partial r}} - \gamma \left( {1 - D} \right)\frac{{\partial T_{D} }}{\partial r}. $$

The constitutive equations with mechanical damage parameter^28^:

5 $$ \sigma_{rr} = \left( {1 - D} \right)\left( {2\mu e_{rr} + \lambda e} \right) - \gamma \left( {1 - D} \right)\left( {T_{D} - T_{0} } \right), $$

6 $$ \sigma_{\psi \psi } = \left( {1 - D} \right)\left( {2\mu e_{\psi \psi } + \lambda e} \right) - \gamma \left( {1 - D} \right)\left( {T_{D} - T_{0} } \right), $$

7 $$ \sigma_{\phi \phi } = \left( {1 - D} \right)\left( {2\mu e_{\phi \phi } + \lambda e} \right) - \gamma \left( {1 - D} \right)\left( {T_{D} - T_{0} } \right), $$

8 $$ \sigma_{r\phi } = \,\,\sigma_{\phi \psi } = \sigma_{r\psi } = 0. $$

The strain components are

9 $$ e_{rr} = \frac{\partial u}{{\partial r}},\quad e_{\psi \psi } = e_{\phi \phi } = \frac{u}{r}, $$

and

10 $$ e_{r\phi } = e_{\phi \psi } = \,e_{r\psi } = 0, $$

where $e$ is the cubical dilatation (volumetric deformation) and satisfies the relation:

11 $$ e = e_{rr} + e_{\psi \psi } + e_{\phi \phi } = \frac{\partial u}{{\partial r}} + \frac{2u}{r} = \frac{1}{{r^{2} }}\frac{{\partial \left( {r^{2} u} \right)}}{\partial r}. $$

The hyperbolic two-temperature heat conduction equations take the forms^7,28^:

12 $$ K\nabla^{2} T_{C} = \rho C_{E} \left( {\frac{\partial }{\partial \,t} + \tau_{0} \frac{{\partial \,^{2} }}{{\partial \,t^{2} }}} \right)T_{D} + \,\gamma T_{0} \left( {\frac{\partial }{\partial \,t} + \tau_{0} \frac{{\partial \,^{2} }}{{\partial \,t^{2} }}} \right)\left( {1 - D} \right)e, $$

and

13 $$ \ddot{T}_{D} = \ddot{T}_{C} - c^{2} \nabla^{2} T_{C} , $$

where $c\left( {m/s} \right)$ is the hyperbolic two-temperature parameter^7^, and $\nabla^{2} = \frac{1}{{r^{2} }}\frac{\partial }{\partial r}\left( {r^{2} \frac{\partial }{\partial r}} \right)$.

We consider that $\varphi = \left( {T_{C} - T_{0} } \right)$ and $\theta = \left( {T_{D} - T_{0} } \right)$ are the conductive and dynamical temperature increment, respectively. Then the equations (4)–(7), (12), and (13) take the forms

14 $$ \rho \left( {\ddot{u} - \Omega^{2} u} \right) = \left( {\lambda + 2\mu } \right)\left( {1 - D} \right)\frac{\partial e}{{\partial r}} - \gamma \left( {1 - D} \right)\frac{\partial \theta }{{\partial r}}, $$

15 $$ \sigma_{rr} = \left( {1 - D} \right)\left( {2\mu e_{rr} + \lambda e} \right) - \gamma \left( {1 - D} \right)\theta , $$

16 $$ \sigma_{\psi \psi } = \left( {1 - D} \right)\left( {2\mu e_{\psi \psi } + \lambda e} \right) - \gamma \left( {1 - D} \right)\theta , $$

17 $$ \sigma_{\phi \phi } = \left( {1 - D} \right)\left( {2\mu e_{\phi \phi } + \lambda e} \right) - \gamma \left( {1 - D} \right)\theta . $$

The equation (14) can be re-written to be in the form

18 $$ \rho \left( {\ddot{e} - \Omega^{2} e} \right) = \left( {\lambda + 2\mu } \right)\left( {1 - D} \right)\nabla^{2} e - \gamma \,\left( {1 - D} \right)\nabla^{2} \theta . $$

For simplicity, we will use the following non-dimensional variables^5,14,16^:

19 $$ \left\{ {r^{\prime},u^{\prime},a^{\prime}} \right\} = c_{o} \eta \,\left\{ {r,u,a} \right\},\,\left\{ {t^{\prime},\tau^{\prime},\tau^{\prime}_{o} ,\tau^{\prime}_{1} } \right\} = c_{o}^{2} \eta \,\left\{ {t,\tau ,\tau_{o} ,\tau_{1} } \right\},\,\,\left\{ {\theta^{\prime},\varphi^{\prime}} \right\} = \frac{1}{{T_{0} }}\left\{ {\theta ,\varphi } \right\},\,\,\sigma^{\prime} = \frac{\sigma }{\mu },\,\,\,\Omega^{\prime} = \frac{\Omega }{{c_{o}^{2} \eta }}. $$

Then, we obtain

20 $$ \,\ddot{e} - \Omega^{2} e = \left( {1 - D} \right)\,\nabla^{2} e - b\left( {1 - D} \right)\nabla^{2} \theta , $$

21 $$ \,\nabla^{2} \varphi = \,\left( {\frac{\partial }{\partial \,t} + \tau_{o} \frac{{\partial \,^{2} }}{{\partial \,t^{2} }}} \right)\theta + \,\varepsilon \left( {\frac{\partial }{\partial \,t} + \tau_{o} \frac{{\partial \,^{2} }}{{\partial \,t^{2} }}} \right)\left( {1 - D} \right)e, $$

22 $$ \ddot{\theta } = \ddot{\varphi } - \tilde{c}^{2} \nabla^{2} \varphi , $$

23 $$ \sigma_{rr} = \left( {1 - D} \right)\left( {\,\beta^{2} e - 2\frac{u}{r}} \right) - \varepsilon_{1} \,\left( {1 - D} \right)\theta , $$

24 $$ \sigma_{\psi \psi } = \left( {1 - D} \right)\left( {\beta^{2} e - 2\frac{\partial \,u}{{\partial \,r}}} \right) - \varepsilon_{1} \,\left( {1 - D} \right)\theta , $$

25 $$ \sigma_{\phi \phi } = \left( {1 - D} \right)\left( {\beta^{2} e - 2\frac{\partial \,u}{{\partial \,r}}} \right) - \varepsilon_{1} \,\left( {1 - D} \right)\theta , $$

where $$\gamma = \left( {3\lambda + 2\mu } \right)\,\alpha_{T}$$,$$c_{o}^{2} = \frac{\lambda + 2\mu }{\rho }$$, $$\eta = \frac{{\rho C_{E} }}{K}$$, $$\varepsilon = \frac{\gamma }{{\rho \,C_{E} }}$$_,_$$\varepsilon_{1} = \frac{{\gamma T_{o} }}{\mu }$$, $$\beta = \left( {\frac{\lambda + 2\mu }{\mu }} \right)^{{{\raise0.7ex\hbox{$1$} \!\mathord{\left/ {\vphantom {1 2}}\right.\kern-\nulldelimiterspace} \!\lower0.7ex\hbox{$2$}}}}$$, $$b = \frac{{\varepsilon_{1} }}{{\beta^{2} }}$$, $$\tilde{c}^{2} = \frac{{c^{2} }}{{c_{o}^{2} }}$$.

The primes have been canceled.

The operator $\nabla^{2} = \frac{1}{{r^{2} }}\frac{\partial }{\partial r}\left( {r^{2} \frac{\partial }{\partial r}} \right)$ is singular at $r = 0$; However, if symmetry conditions prevail, the singularity situation is reduced by applying L’Hopital’s rule as follows^18^:

$$ \nabla^{2} \left\{ {e,\theta ,\varphi } \right\} = \mathop {\lim }\limits_{r \to 0} \left[ {\frac{1}{{r^{2} }}\frac{\partial }{\partial r}\left( {r^{2} \frac{{\partial \left\{ {e,\theta ,\varphi } \right\}}}{\partial r}} \right)} \right] = \mathop {\lim }\limits_{r \to 0} \left[ {\frac{{\partial^{2} \left\{ {e,\theta ,\varphi } \right\}}}{{\partial r^{2} }} + \frac{2}{r}\frac{{\partial \left\{ {e,\theta ,\varphi } \right\}}}{\partial r}} \right] = \frac{{\partial^{2} \left\{ {e,\theta ,\varphi } \right\}}}{{\partial r^{2} }} + 2\frac{{\partial^{2} \left\{ {e,\theta ,\varphi } \right\}}}{{\partial r^{2} }}. $$

Then, we get

26 $$ \nabla^{2} \left\{ {e,\theta ,\varphi } \right\} = 3\frac{{\partial^{2} }}{{\partial r^{2} }}\,\left\{ {e,\theta ,\varphi } \right\}, $$

and satisfy the boundary conditions

27 $$ \left. {\frac{\partial }{\partial r}\left\{ {e,\theta ,\varphi } \right\}} \right|_{r = 0} = 0. $$

Hence, we have

28 $$ \nabla^{2} e\left( {r,t} \right) = 3\frac{{\partial^{2} e\left( {r,t} \right)}}{{\partial r^{2} }},\,\,\,\nabla^{2} \theta \left( {r,t} \right) = 3\frac{{\partial^{2} \theta \left( {r,t} \right)}}{{\partial r^{2} }},\,\nabla^{2} \varphi \left( {r,t} \right) = 3\frac{{\partial^{2} \varphi \left( {r,t} \right)}}{{\partial r^{2} }}. $$

By applying the forms (26) in equations (20)–(22), we obtain

29 $$ \ddot{e} - \Omega^{2} e = 3\left( {1 - D} \right)\,\frac{{\partial^{2} e}}{{\partial r^{2} }} - 3b\left( {1 - D} \right)\frac{{\partial^{2} \theta }}{{\partial r^{2} }}, $$

30 $$ \,3\frac{{\partial^{2} \varphi }}{{\partial r^{2} }} = \left( {\frac{\partial }{\partial \,t} + \tau_{o} \frac{{\partial \,^{2} }}{{\partial \,t^{2} }}} \right)\theta + \,\varepsilon \left( {\frac{\partial }{\partial \,t} + \tau_{o} \frac{{\partial \,^{2} }}{{\partial \,t^{2} }}} \right)\left( {1 - D} \right)e, $$

31 $$ \ddot{\theta } = \ddot{\varphi } - 3\tilde{c}^{2} \frac{{\partial^{2} \varphi }}{{\partial r^{2} }}. $$

We apply the Laplace transform, which is defined as:

32 $$ \ell \left\{ {f\left( t \right)} \right\} = \overline{f}\left( s \right) = \int_{0}^{\infty } {f\left( t \right)\,e^{ - st} dt} , $$

and

33 $$ \left. {\frac{{\partial \theta \left( {r,t} \right)}}{\partial t}} \right|_{t = 0} = \left. {\frac{{\partial \varphi \left( {r,t} \right)}}{\partial t}} \right|_{t = 0} = \left. {\frac{{\partial e\left( {r,t} \right)}}{\partial t}} \right|_{t = 0} = 0. $$

Thus, the equations (20)–(25) have the forms

34 $$ \left( {s^{2} - \Omega^{2} } \right)\overline{e} = 3\left( {1 - D} \right)\,\frac{{\partial^{2} \overline{e}}}{{\partial r^{2} }} - 3b\left( {1 - D} \right)\frac{{\partial^{2} \overline{\theta }}}{{\partial r^{2} }}, $$

35 $$ 3\frac{{\partial^{2} \overline{\varphi }}}{{\partial r^{2} }} = \,\left( {s + \tau_{o} s^{2} } \right)\overline{\theta } + \,\varepsilon \left( {s + \tau_{o} s^{2} } \right)\left( {1 - D} \right)\,\overline{e}, $$

36 $$ \overline{\theta } = \overline{\varphi } - \delta^{2} \frac{{\partial^{2} \overline{\varphi }}}{{\partial r^{2} }}, $$

37 $$ \overline{\sigma }_{rr} = \left( {1 - D} \right)\left( {\,\beta^{2} \overline{e} - 2\frac{{\overline{u}}}{r}} \right) - \varepsilon_{1} \left( {1 - D} \right)\,\overline{\theta }, $$

38 $$ \overline{\sigma }_{\psi \psi } = \left( {1 - D} \right)\left( {\beta^{2} \overline{e} - 2\frac{{\partial \,\overline{u}}}{\partial \,r}} \right) - \varepsilon_{1} \left( {1 - D} \right)\overline{\theta }, $$

39 $$ \overline{\sigma }_{\phi \phi } = \left( {1 - D} \right)\left( {\beta^{2} \overline{e} - 2\frac{{\partial \,\overline{u}}}{\partial \,r}} \right) - \varepsilon_{1} \left( {1 - D} \right)\overline{\theta }, $$

40 $$ \overline{e} = \frac{1}{{r^{2} }}\frac{{\partial \left( {r^{2} \overline{u}} \right)}}{\partial r}, $$

where $$\delta^{2} = \frac{{3\tilde{c}^{2} }}{{s^{2} }}$$.

Substitute from equation (36) into equations (34) and (35), we get

41 $$ \left( {\frac{{\partial^{2} }}{{\partial r^{2} }} - \alpha_{1} } \right)\overline{e} = b\frac{{\partial^{2} \overline{\varphi }}}{{\partial r^{2} }}\, - \delta^{2} b\frac{{\partial^{4} \overline{\varphi }}}{{\partial r^{4} }}, $$

42 $$ \frac{{\partial^{2} \overline{\varphi }}}{{\partial r^{2} }} = \,\alpha_{2} \overline{\varphi } + \alpha_{3} \,\overline{e}, $$

where $$\alpha_{1} = \frac{{s^{2} - \Omega^{2} }}{{3\left( {1 - D} \right)}}$$ , $$\alpha_{2} = \frac{{s + \tau_{o} s^{2} }}{{3 + \delta^{2} \left( {s + \tau_{o} s^{2} } \right)}}$$ and $$\alpha_{3} = \frac{{\varepsilon \,\left( {s + \tau_{o} s^{2} } \right)\left( {1 - D} \right)\,}}{{3 + \delta^{2} \left( {s + \tau_{o} s^{2} } \right)}}$$.

Substituting from equation (42) into the equation (41), we obtain

43 $$ \,\frac{{\partial^{2} \overline{e}}}{{\partial r^{2} }} = \alpha_{4} \overline{\varphi } + \alpha_{5} \overline{e}, $$

where $$\alpha_{4} = \frac{{b\alpha_{2} \left( {1 - \delta^{2} \alpha_{2} } \right)}}{{1 + \delta^{2} b\alpha_{3} }}$$ and $$\,\alpha_{5} = \frac{{\alpha_{1} + b\alpha_{3} \left( {\,1\, - \delta^{2} \alpha_{2} } \right)}}{{1 + \delta^{2} b\alpha_{3} }}$$.

## The diagonalization method

We can re-write the equations (42) and (43) in a matrix form as follows^29^:

44 $$ \frac{d}{dr}\left[ {\begin{array}{*{20}c} {\overline{\varphi }} \\ {\overline{e}} \\ {\overline{\varphi }^{\prime}} \\ {\overline{e}^{\prime}} \\ \end{array} } \right] = \left[ {\begin{array}{*{20}c} 0 & 0 & 1 & 0 \\ 0 & 0 & 0 & 1 \\ {\alpha_{2} } & {\alpha_{3} } & 0 & 0 \\ {\alpha_{4} } & {\alpha_{5} } & 0 & 0 \\ \end{array} } \right]\left[ {\begin{array}{*{20}c} {\overline{\varphi }} \\ e \\ {\overline{\varphi }^{\prime}} \\ {\overline{e}^{\prime}} \\ \end{array} } \right]. $$

For simplicity, we write the system in (44) as a homogenous system of linear first-order differential equation as^29^:

45 $$ \frac{dZ\left( r \right)}{{dr}} = AZ\left( r \right), $$

where $$Z\left( r \right) = \left[ {\begin{array}{*{20}c} {\overline{\varphi }\left( r \right)} \\ {\overline{e}\left( r \right)} \\ {\overline{\varphi }^{\prime}\left( r \right)} \\ {\overline{e}^{\prime}\left( r \right)} \\ \end{array} } \right]$$ and $$A = \left[ {\begin{array}{*{20}c} 0 & 0 & 1 & 0 \\ 0 & 0 & 0 & 1 \\ {\alpha_{2} } & {\alpha_{3} } & 0 & 0 \\ {\alpha_{4} } & {\alpha_{5} } & 0 & 0 \\ \end{array} } \right]$$.

The matrix *A* has four linearly independent eigenvectors; hence, we can construct a matrix *V* from the eigenvectors of the matrix *A* such that $V^{ - 1} AV = W$ where $W$ is a diagonal matrix^29^.

If we make the substitution $Z = V\,Y$ in the system (45), then

46 $$ V\,Y^{\prime} = AV\,Y\,\,{\text{or}}\,\,\,\,Y^{\prime} = V^{ - 1} AV\,Y = W\,Y, $$

which gives

47 $$ \left[ {\begin{array}{*{20}c} {y^{\prime}_{1} } \\ {y^{\prime}_{2} } \\ {y^{\prime}_{3} } \\ {y^{\prime}_{4} } \\ \end{array} } \right] = \left[ {\begin{array}{*{20}c} {\lambda_{1} } & 0 & 0 & 0 \\ 0 & {\lambda_{2} } & 0 & 0 \\ 0 & 0 & {\lambda_{3} } & 0 \\ 0 & 0 & 0 & {\lambda_{4} } \\ \end{array} } \right]\left[ {\begin{array}{*{20}c} {y_{1} } \\ {y_{2} } \\ {y_{3} } \\ {y_{4} } \\ \end{array} } \right], $$

where $\pm \lambda_{1} {\text{and}}\, \pm \lambda_{2}$ are the eigenvalues of the matrix *A* or the roots of the characteristic equation

48 $$ \lambda^{4} - L\,\lambda^{2} + M = 0, $$

where

49 $$ L = \lambda_{1}^{2} + \lambda_{2}^{2} = \alpha_{2} + \alpha_{5} ,\,\,\,M = \lambda_{1}^{2} \lambda_{2}^{2} = \alpha_{2} \alpha_{5} - \alpha_{3} \alpha_{4} ,\,\,\,\lambda_{2} = - \lambda_{1} ,\,\,\,\,\lambda_{4} = - \lambda_{3} . $$

Since *W* is a diagonal matrix, then the system (47) is uncoupled, making each differential equation in the system has the form $y_{i}^{\prime } = \lambda_{i} y_{i} ,\,\,i = 1,2,3,4$. The solution to each of these linear equations is $y_{i} = c_{i} e^{{\lambda_{i} x}} ,\,\,i = 1,2,3,4$. Hence, the general solution of the system (47) can be written as column vector^29^:

50 $$ Y = \left[ {\begin{array}{*{20}c} {c_{1} e^{{\lambda_{1} r}} } \\ {c_{2} e^{{ - \lambda_{1} r}} } \\ {c_{3} e^{{\lambda_{2} r}} } \\ {c_{4} e^{{ - \lambda_{2} r}} } \\ \end{array} } \right]. $$

Then the final solution of the system (45) is

51 $$ Z\left( r \right) = V\,Y\left( r \right). $$

The matrix *V* from the eigenvectors of the matrix *A* takes the form

52 $$ V = \left[ {\begin{array}{*{20}c} {\frac{{\alpha_{3} }}{{\lambda_{1} \left( {\lambda_{1}^{2} - \alpha_{2} } \right)}}} & {\frac{{ - \alpha_{3} }}{{\lambda_{1} \left( {\lambda_{1}^{2} - \alpha_{2} } \right)}}} & {\frac{{\alpha_{3} }}{{\lambda_{2} \left( {\lambda_{2} - \alpha_{2} } \right)}}} & {\frac{{ - \alpha_{3} }}{{\lambda_{2} \left( {\lambda_{2} - \alpha_{2} } \right)}}} \\ {\frac{1}{{\lambda_{1} }}} & { - \frac{1}{{\lambda_{1} }}} & {\frac{1}{{\lambda_{2} }}} & { - \frac{1}{{\lambda_{2} }}} \\ {\frac{{\alpha_{3} }}{{\left( {\lambda_{1}^{2} - \alpha_{2} } \right)}}} & {\frac{{\alpha_{3} }}{{\left( {\lambda_{1}^{2} - \alpha_{2} } \right)}}} & {\frac{{\alpha_{3} }}{{\left( {\lambda_{2} - \alpha_{2} } \right)}}} & {\frac{{\alpha_{3} }}{{\left( {\lambda_{2} - \alpha_{2} } \right)}}} \\ 1 & 1 & 1 & 1 \\ \end{array} } \right]. $$

Substitute from Eqs. (50) and (52) into the Eq. (51), we get

53 $$ \left[ {\begin{array}{*{20}c} {\overline{\varphi }\left( r \right)} \\ {\overline{e}\left( r \right)} \\ {\overline{\varphi }^{\prime}\left( r \right)} \\ {\overline{e}^{\prime}\left( r \right)} \\ \end{array} } \right] = \left[ {\begin{array}{*{20}c} {\frac{{\alpha_{3} }}{{\lambda_{1} \left( {\lambda_{1}^{2} - \alpha_{2} } \right)}}} & {\frac{{ - \alpha_{3} }}{{\lambda_{1} \left( {\lambda_{1}^{2} - \alpha_{2} } \right)}}} & {\frac{{\alpha_{3} }}{{\lambda_{2} \left( {\lambda_{2} - \alpha_{2} } \right)}}} & {\frac{{ - \alpha_{3} }}{{\lambda_{2} \left( {\lambda_{2} - \alpha_{2} } \right)}}} \\ {\frac{1}{{\lambda_{1} }}} & { - \frac{1}{{\lambda_{1} }}} & {\frac{1}{{\lambda_{2} }}} & { - \frac{1}{{\lambda_{2} }}} \\ {\frac{{\alpha_{3} }}{{\left( {\lambda_{1}^{2} - \alpha_{2} } \right)}}} & {\frac{{\alpha_{3} }}{{\left( {\lambda_{1}^{2} - \alpha_{2} } \right)}}} & {\frac{{\alpha_{3} }}{{\left( {\lambda_{2} - \alpha_{2} } \right)}}} & {\frac{{\alpha_{3} }}{{\left( {\lambda_{2} - \alpha_{2} } \right)}}} \\ 1 & 1 & 1 & 1 \\ \end{array} } \right]\left[ {\begin{array}{*{20}c} {c_{1} e^{{\lambda_{1} r}} } \\ {c_{2} e^{{ - \lambda_{1} r}} } \\ {c_{3} e^{{\lambda_{2} r}} } \\ {c_{4} e^{{ - \lambda_{2} r}} } \\ \end{array} } \right]. $$

The boundary conditions in (27) and equation (53) give that

54 $$ c_{1} = - c_{2} \,{\text{and}}\,\,c_{3} = - c_{4} . $$

Hence, we obtain

55 $$ \overline{\varphi }\,\left( {r,s} \right) = \,\alpha_{3} \sum\limits_{i = 1}^{2} {\frac{{A_{i} \cosh \left( {\lambda_{i} r} \right)}}{{\lambda_{i} \left( {\lambda_{i}^{2} - \alpha_{2} } \right)}}} , $$

and

56 $$ \overline{e}\,\left( {r,s} \right) = \sum\limits_{i = 1}^{2} {\frac{{A_{i} \cosh \left( {\lambda_{i} r} \right)}}{{\lambda_{i} }}} . $$

To get the constants $A_{1} \,{\text{and}}\,A_{2}$, we must apply the boundary conditions at $r = a$; we consider the sphere when $r = a$ is thermally shocked as follows:

57 $$ \varphi \left( {a,t} \right) = \varphi_{o} H\left( t \right), $$

where $H\left( t \right)$ is called the Heaviside unit step function and $\varphi_{o}$ is constant, which gives the strength of the thermal shock.

Moreover, we consider the bounding surface of the sphere $r = a$ is connected to a rigid foundation that can prevent any displacement. Thus, no volumetric deformation in the bounding surface of the sphere as follows:

58 $$ e\left( {a,t} \right) = 0. $$

Applying Laplace transform to the Eqs. (57) and (58), we get

59 $$ \overline{\varphi }\left( {a,s} \right) = \frac{{\varphi_{o} }}{s}, $$

and

60 $$ \overline{e}\left( {a,s} \right) = 0. $$

Applying the boundary conditions to the Eqs. (55) and (56), we obtain the following system of equations:

61 $$ \sum\limits_{i = 1}^{2} {\frac{{A_{i} \cosh \left( {\lambda_{i} a} \right)}}{{\lambda_{i} \left( {\lambda_{i}^{2} - \alpha_{2} } \right)}}} = \frac{{\varphi_{0} }}{{s\alpha_{3} \,}}, $$

and

62 $$ \sum\limits_{i = 1}^{2} {\frac{{A_{i} \cosh \left( {\lambda_{i} a} \right)}}{{\lambda_{i} }}} = 0. $$

By solving the system (61) and (62) using the relations between the roots (49), we get

$$A_{1} = \frac{{\varphi_{0} \alpha_{4} \lambda_{1} }}{{s\left( {\lambda_{1}^{2} - \lambda_{2}^{2} } \right)\cosh \left( {\lambda_{1} a} \right)}}$$ and $$A_{2} = - \frac{{\varphi_{0} \alpha_{4} \lambda_{2} }}{{s\left( {\lambda_{1}^{2} - \lambda_{2}^{2} } \right)\cosh \left( {\lambda_{2} a} \right)}}$$.

Hence, we have

63 $$ \overline{\varphi }\,\left( {r,s} \right) = \,\frac{{\varphi_{0} \alpha_{3} \alpha_{4} }}{{s\left( {\lambda_{1}^{2} - \lambda_{2}^{2} } \right)}}\left[ {\frac{{\cosh \left( {\lambda_{1} r} \right)}}{{\left( {\lambda_{1}^{2} - \alpha_{2} } \right)\cosh \left( {\lambda_{1} a} \right)}} - \frac{{\cosh \left( {\lambda_{2} r} \right)}}{{\left( {\lambda_{2}^{2} - \alpha_{2} } \right)\cosh \left( {\lambda_{2} a} \right)}}} \right], $$

and

64 $$ \overline{e}\,\left( {r,s} \right) = \frac{{\varphi_{0} \alpha_{4} }}{{s\left( {\lambda_{2}^{2} - \lambda_{1}^{2} } \right)}}\left[ {\frac{{\cosh \left( {\lambda_{1} r} \right)}}{{\cosh \left( {\lambda_{1} a} \right)}} - \frac{{\cosh \left( {\lambda_{2} r} \right)}}{{\cosh \left( {\lambda_{2} a} \right)}}} \right]. $$

To obtain the displacement function, we can use the Eqs. (40) and (64) as follows:

65 $$ \overline{u}\left( {r,s} \right) = \frac{{\int {r^{2} \overline{e}\left( {r,s} \right)\,} \partial r}}{{r^{2} }}. $$

The singularity situation problem (65) can be reduced by using L’Hopital’s rule again as follows^18^:

66 $$ \overline{u}\left( {r,s} \right) = \mathop {\lim }\limits_{r \to 0} \frac{{\int {\left( {r^{2} \overline{e}\left( {r,s} \right)} \right)\,} \partial r}}{{r^{2} }} = \mathop {\lim }\limits_{r \to 0} \frac{{r^{2} \overline{e}\left( {r,s} \right)}}{2r} = \frac{{r\overline{e}\left( {r,s} \right)}}{2}. $$

Hence, we have

67 $$ \overline{u}\left( {r,s} \right) = \frac{{\varphi_{0} \alpha_{4} r}}{{2\,s\left( {\lambda_{2}^{2} - \lambda_{1}^{2} } \right)}}\left[ {\frac{{\cosh \left( {\lambda_{1} r} \right)}}{{\cosh \left( {\lambda_{1} a} \right)}} - \frac{{\cosh \left( {\lambda_{2} r} \right)}}{{\cosh \left( {\lambda_{2} a} \right)}}} \right]. $$

To obtain the stress function in a simple form, we take the average of the three principal stresses components on (37)–(39) to be as follows:

68 $$ \overline{\sigma }\left( {r,s} \right) = \frac{{\overline{\sigma }_{rr} + \overline{\sigma }_{\psi \psi } + \overline{\sigma }_{\phi \phi } }}{3} = \left( {\beta^{2} - 4/3} \right)\left( {1 - D} \right)\overline{e}\left( {r,s} \right) - \varepsilon_{1} \left( {1 - D} \right)\overline{\theta }\left( {r,s} \right). $$

**Figure 2:**
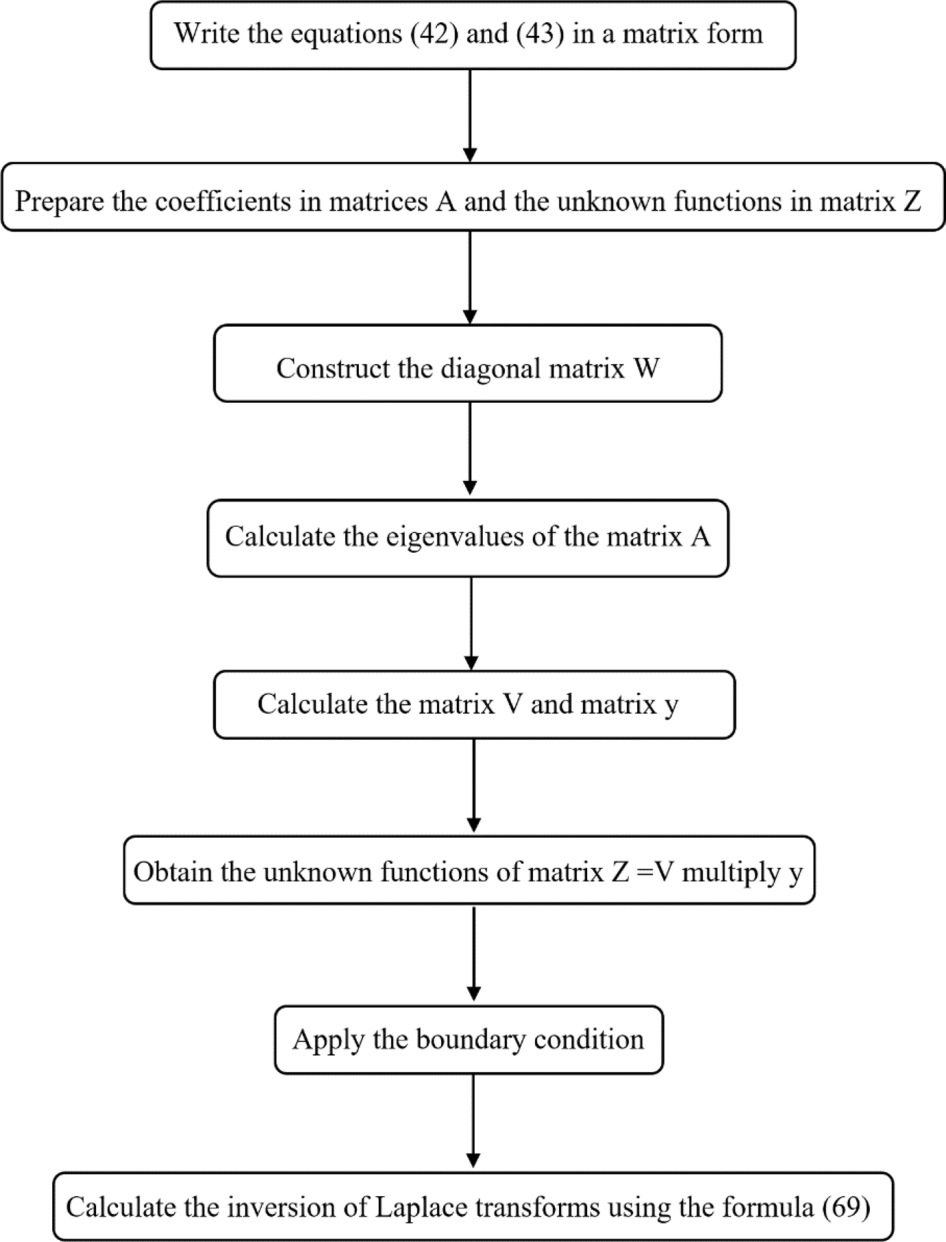
The flowchart of the method.

The Riemann-sum approximation techniques will be used to compute the studied functions' numerical solutions in the time domain. By this method, the Laplace transform of any function can be inverted as^30^:

69 $$ f(t) = \frac{{e^{\kappa t} }}{t}\left[ {\frac{1}{2}\overline{f}\left( \kappa \right) + {\text{Re}} \sum\limits_{n = 1}^{N} {\left( { - 1} \right)^{n} \overline{f}\left( {\kappa + \frac{i\,n\pi }{t}} \right)} } \right], $$

where “$i$” is the imaginary number unit, *“Re”* is the real part, and the value $\kappa$ satisfies the relation $\kappa \,t \approx 4.7$^30^. Figure 2 is the flowchart which represents the method and all steps.

After getting the stress and strain functions in the original time-domain, we can obtain the stress–strain energy as follows^31^:

70 $$ \varpi \left( {r,t} \right) = \frac{1}{2}\sigma_{ij} \left( {r,t} \right)e_{ij} \left( {r,t} \right). $$

For the present application, the stress–strain energy takes the form

71 $$ \varpi \left( {r,t} \right) = \frac{1}{2}\left( {\sigma_{rr} e_{rr} + \sigma_{\psi \psi } e_{\psi \psi } + \sigma_{\phi \phi } e_{\phi \phi } } \right). $$

After eliminating the term with a small value, we get

72 $$ \varpi \left( {r,t} \right) \approx \frac{1}{2}\left( {1 - D} \right)\left[ {\beta^{2} e^{2} \left( {r,t} \right) - \varepsilon_{1} \,e\,\left( {r,t} \right)\theta \left( {r,t} \right)} \right]. $$

## Numerical results and discussion

To obtain the numerical results, the copper material has been taken as the thermoelastic material for which we use the following values of the material properties^11^:

$$K = 386\,\,{\text{kg}}\,{\text{m}}\,{\text{k}}^{ - 1} {\text{s}}^{ - 3} \,$$, $$C_{E} = 383.1\,\,\,{\text{m}}^{2} \,{\text{k}}^{ - 1} \,{\text{s}}^{ - 2}$$, $$\alpha_{T} = 1.78\;\left( {10} \right)^{ - 5} \,{\text{k}}^{ - 1}$$, $$T_{o} = 293\,{\text{k}}$$, $$\rho = 8954\,\,{\text{kg}}\,{\text{m}}^{ - 3}$$, $$\mu = 3.86\;\left( {10} \right)^{10} \,{\text{kg}}\,\,{\text{m}}^{ - 1} \,{\text{s}}^{ - 2}$$, $$\lambda = 7.76\;\left( {10} \right)^{10} {\text{kg}}\,\,{\text{m}}^{ - 1} \,{\text{s}}^{ - 2}$$.

Thus, we have the following non-dimensional values of parameters:

$$b = 0.01047$$, $$\varepsilon_{1} = {0}{\text{.0419,}}\,\,\,\varepsilon = 1.6086$$, $$\beta^{2} = 4$$, $$\varphi_{0} = 1.0$$, $$\tau_{o} = 0.02$$, $$\tau_{o} = 0.01$$.

The numerical results of the conductive and dynamical temperature increments, strain, displacement, average stress, and stress–strain energy distributions have been represented in figures with a wide range of non-dimensional radial distance $r\left( {0 \le r \le 5.0} \right)$ at the non-dimension instant of time $t = 1.0$.

**Figure 3:**
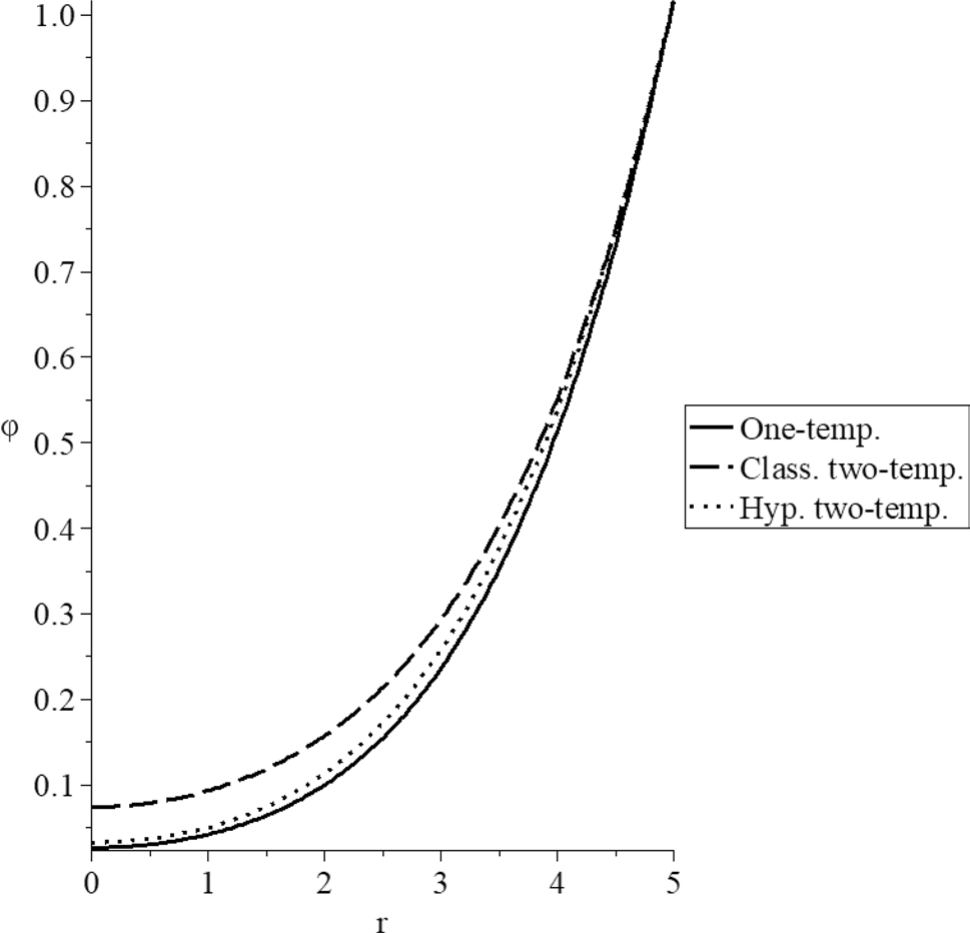
The conductive temperature increment distribution for different models.

**Figure 4:**
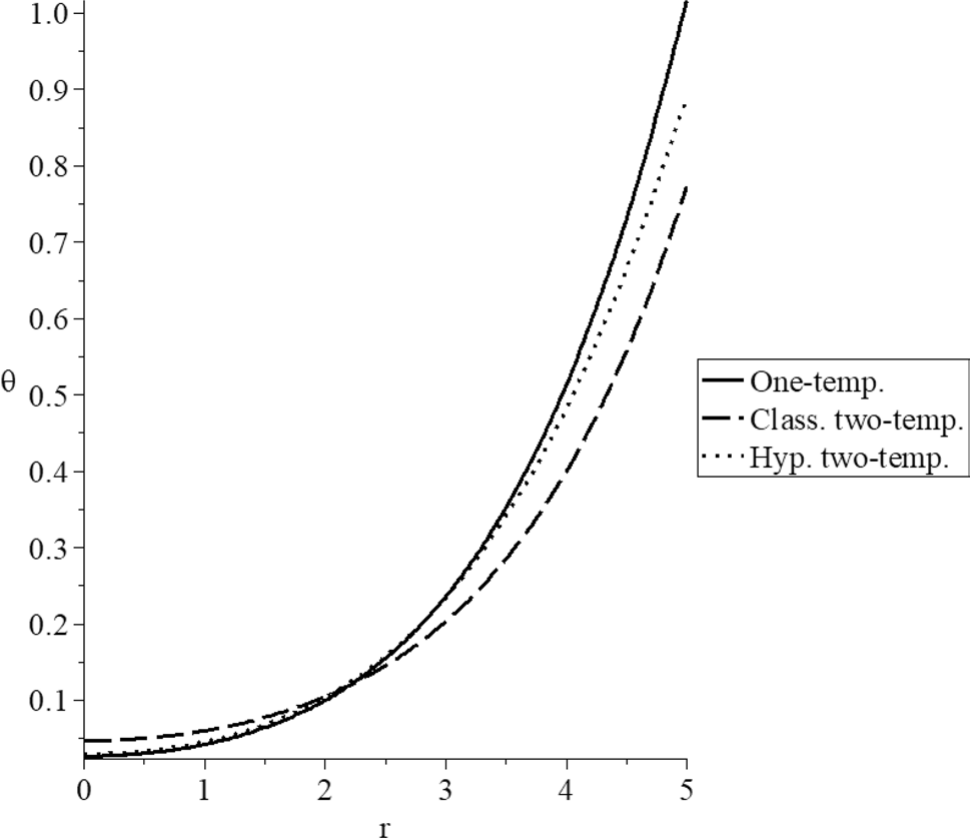
The dynamical temperature increment distribution for different models.

**Figure 5:**
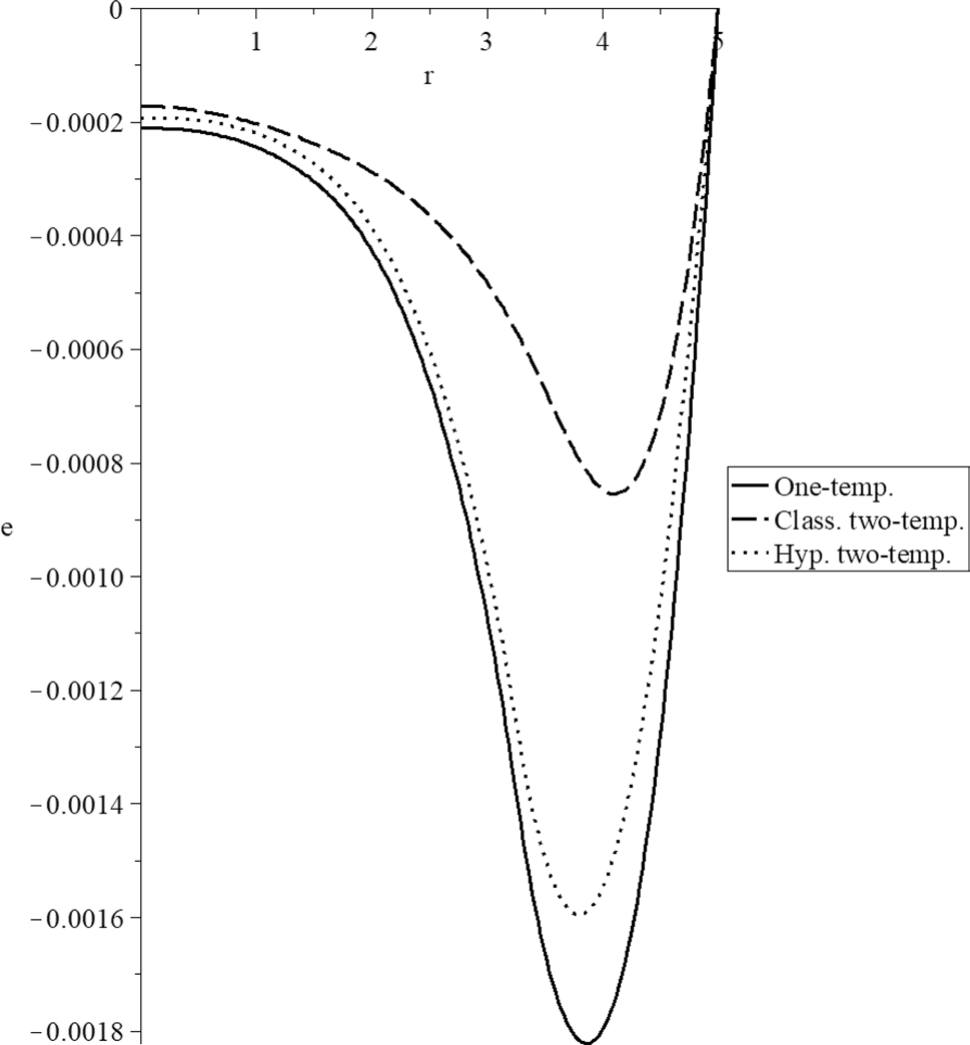
The volumetric deformation distribution for different models.

**Figure 6:**
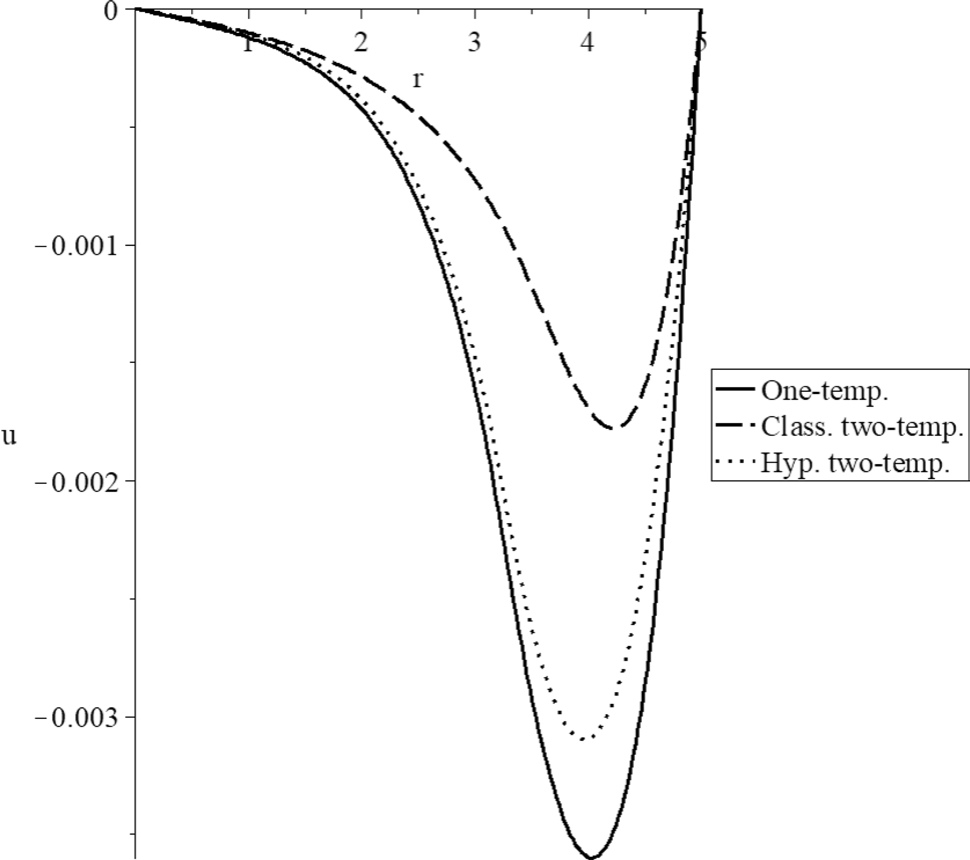
The displacement distribution for different models.

**Figure 7:**
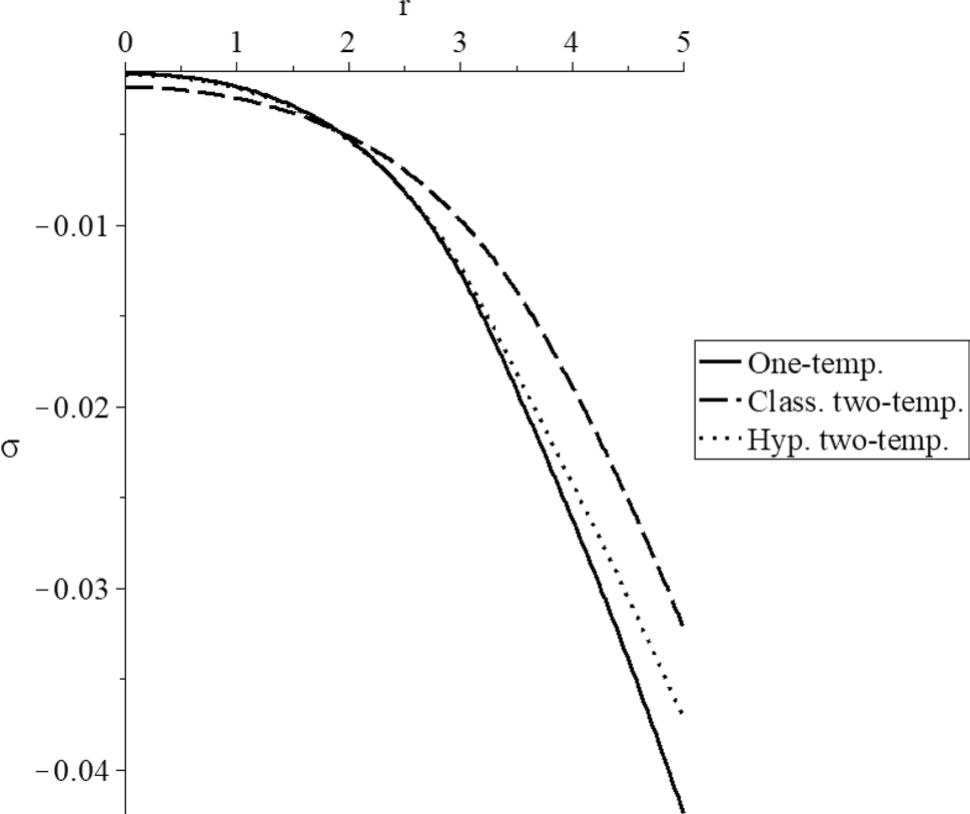
The average stress distribution for different models.

**Figure 8:**
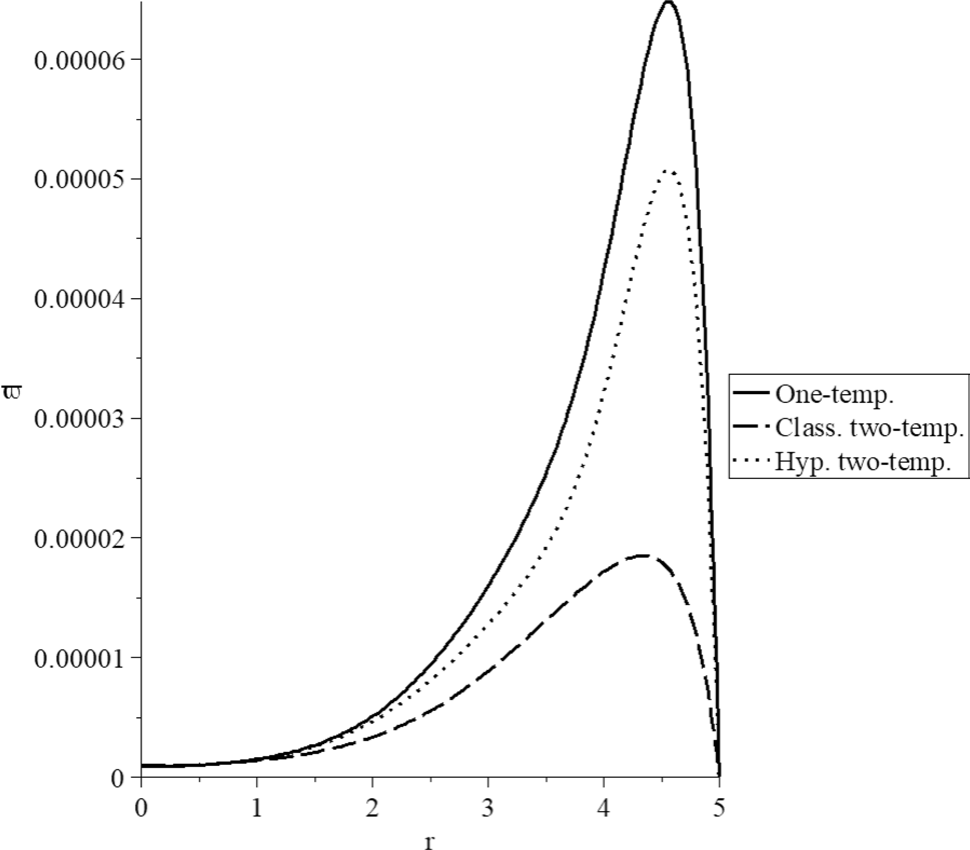
The stress-strain energy distribution for different models.

Figures 3, 4, 5, 6, 7 and 8 have been carried out for various values of the two-temperature parameter $\tilde{c} = \left( {0.0,0.5} \right)$, which gives $\delta^{2} = 3\tilde{c}^{2} /s^{2} = \left( {0.0,3\left( {0.5} \right)^{2} ,3\left( {0.5/s} \right)^{2} } \right)$, where the value $\delta = 0.0$ represents the L–S model of one-temperature, it has been figured in solid curves. The value $\tilde{c} = 0.5$ represents two cases; the first case is $\delta^{2} = 3\left( {0.5} \right)^{2}$ which represents the classical two-temperature model and has been figured with dash curves, while the second case is $\delta^{2} = 3\left( {\frac{0.5}{s}} \right)^{2}$ which represents the hyperbolic two-temperature model and has been figured in dote curves. The numerical results of those figures have been calculated when the mechanical damage parameter $D = 0.0$ and rotation parameter $\Omega = 0.0$.

Figure 3 shows the conductive temperature increment distributions. It is noted that all the curves start from the position $r = 5.0$ with the same value $\varphi \left( {r = 5.0} \right) = 1.0$ as the boundary condition on the surface of the sphere. The three curves have the same behavior but with different values. The two-temperature parameter has a significant impact on the conductive temperature increment distribution. In the center of the sphere, the conductive temperature increment values based on the one-temperature and hyperbolic two-temperature vanish before its values in the classical two-temperature model. Therefore, the thermal wave due to the conductive temperature propagates with a finite speed in the context of one-temperature and hyperbolic two-temperature models. In contrast, it propagates with infinite speed in the context of the classical two-temperature model.

Figure 4 represents the dynamical temperature increment distributions. It is noted that the three curves start from the position $r = 5.0$ with different values $\theta \left( {{\text{One - temp}}{.}} \right) = 1.0$, which is the value of the thermal shock on the bounding surface of the sphere $\theta \left( {{\text{Class}}{.}\,{\text{two - temp}}{.}} \right) = 0.98$, and $\theta \left( {{\text{Hyp}}{.}\,{\text{two - temp}}{.}} \right) = 0.75$. The three curves have the same behavior but have different values. The two-temperature parameter has a significant effect on the dynamical temperature distribution. In the center of the sphere, the values of dynamical temperature increment in the context of the one-temperature and hyperbolic two-temperature vanish before its values in the classical two-temperature model. It means that the dynamical thermal wave propagates with a finite speed in the context of one-temperature and hyperbolic two-temperature models. In contrast, it propagates with infinite speed in the context of the classical two-temperature model.

Figure 5 represents the volumetric strain distributions. It is noted that the three curves start from the position $r = 5.0$ with the zero value $e\left( {r = 5.0} \right) = 0.0$ as the boundary condition on the bounding surface of the sphere. The three curves have the same behavior but have different values. Each curve has one peak point, and the absolute values of the peak points take the following order:

73 $$ \left| {e_{\max } \left( {{\text{One - temp}}.} \right)} \right| > \left| {e_{\max } \left( {{\text{Hyp}}{.}\,\,{\text{two - temp}}.} \right)} \right| > \left| {e_{\max } \left( {{\text{Class}}{.}\,\,{\text{two - temp}}.} \right)} \right| $$

Figure 6 shows the displacement distribution. The three curves start from the position $r = 5.0$ with zero value $u\left( {r = 5.0} \right) = 0.0$. The three curves have the same behavior but have different values. Each curve has a peak point, and the absolute values of the peak points take the following order:

74 $$ \left| {u_{\max } \left( {{\text{One - temp}}.} \right)} \right| > \left| {u_{\max } \left( {{\text{Hyp}}{.}\,\,{\text{two - temp}}.} \right)} \right| > \left| {u_{\max } \left( {{\text{Class}}{.}\,\,{\text{two - temp}}.} \right)} \right| $$

Figure 7 shows the average stress distribution, and it is noted that the three curves start from the position $r = 5.0$ with different values. All the curves have the same behavior with different values. In the center of the sphere, stress values in the context of the one-temperature and hyperbolic two-temperature vanish before its values in the classical two-temperature model. It means that the mechanical wave propagates with a finite speed in the context of one-temperature and hyperbolic two-temperature models. In contrast, it propagates with infinite speed in the context of the classical two-temperature model.

Figure 8 represents the stress–strain energy distribution, and it is noted that the three curves start from the position $r = 5.0$ with the zero value. The three curves have the same behavior and different values. Each curve has one peak point, and the values of the peak point take the following order:

75 $$ \varpi_{\max } \left( {{\text{One - temp}}.} \right) > \varpi_{\max } \left( {{\text{Hyp}}{.}\,\,{\text{two - temp}}.} \right) > \varpi_{\max } \left( {{\text{Class}}{.}\,\,{\text{two - temp}}.} \right) $$

**Figure 9:**
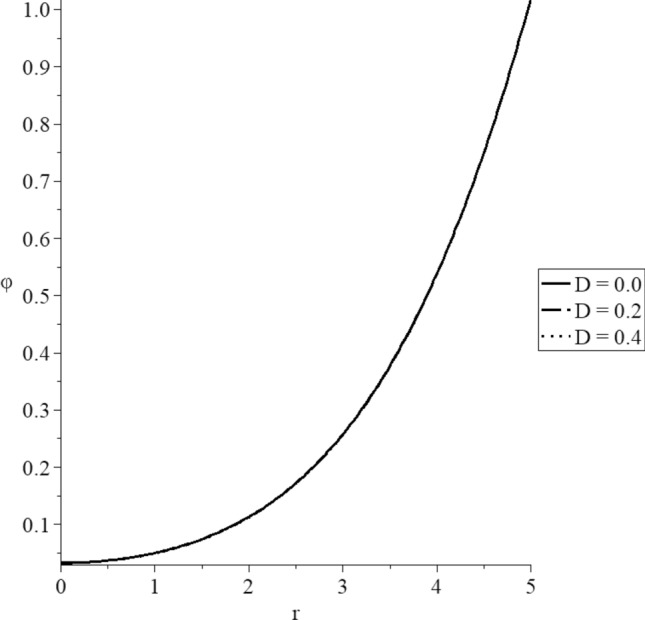
The conductive temperature increment distribution with various values of the mechanical damage parameter.

**Figure 10:**
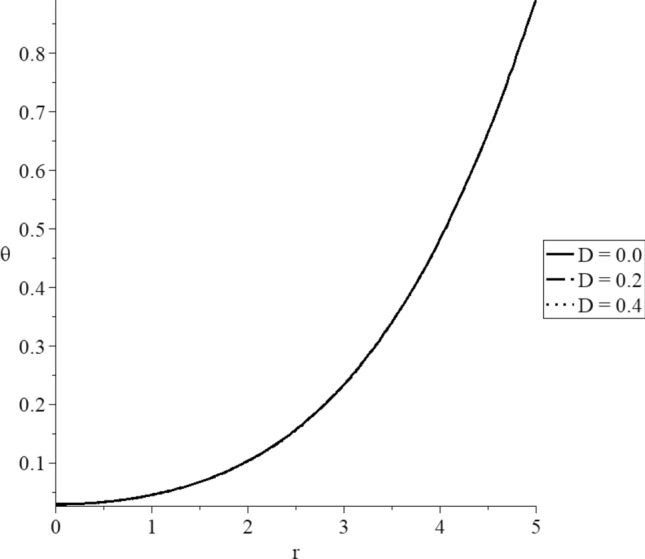
The dynamical temperature increment distribution with various values of the mechanical damage parameter.

**Figure 11:**
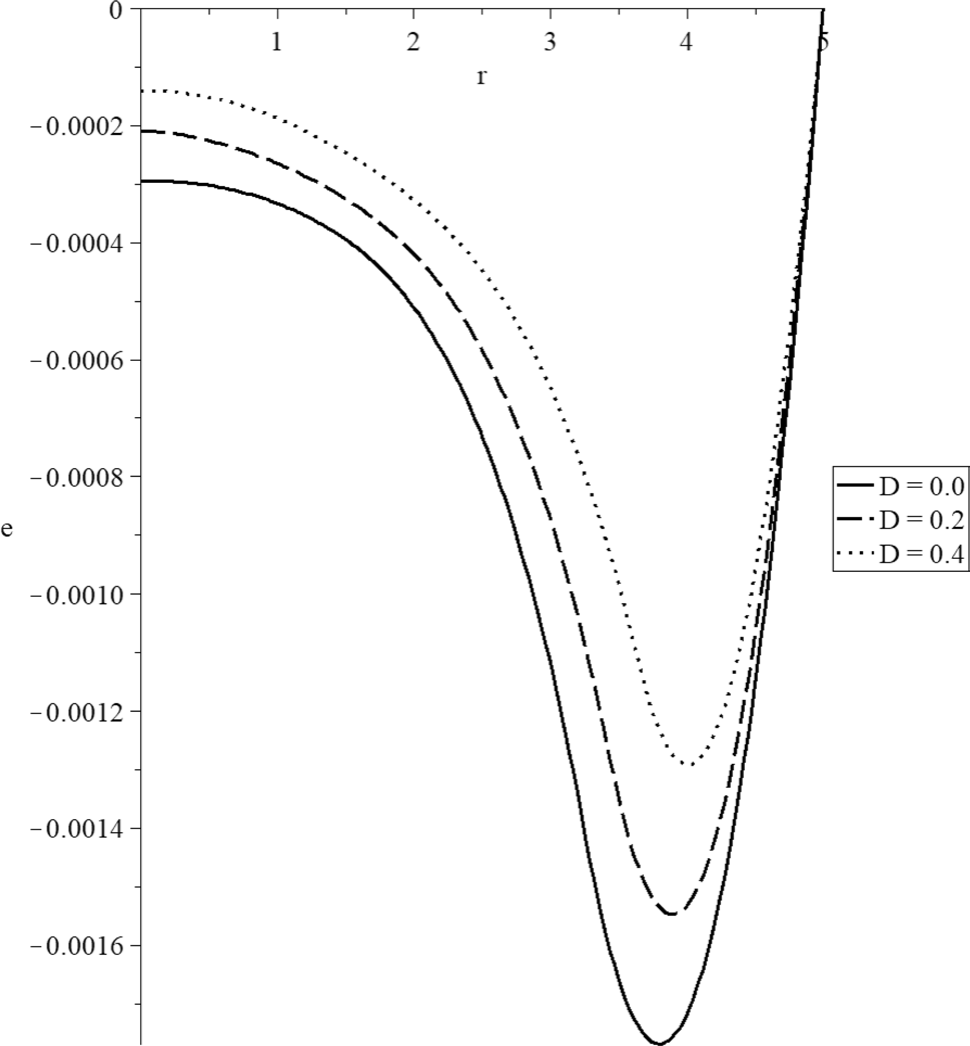
The volumetric deformation distribution with various values of the mechanical damage parameter.

**Figure 12:**
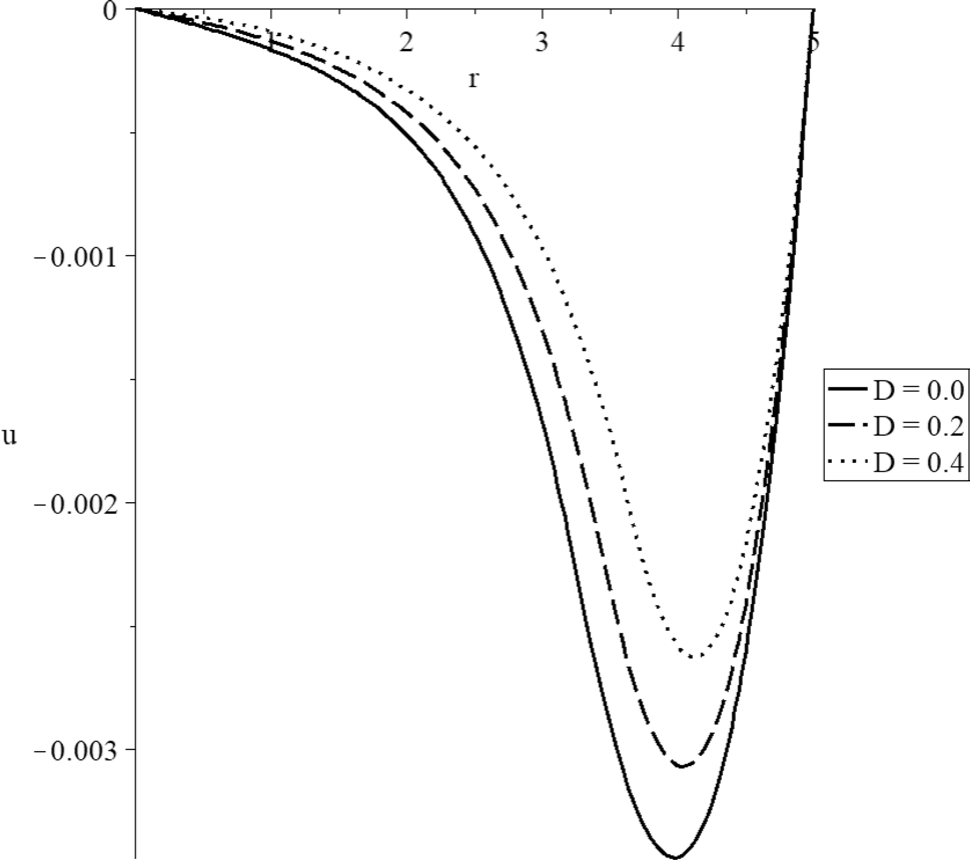
The displacement distribution with various values of the mechanical damage parameter.

**Figure 13:**
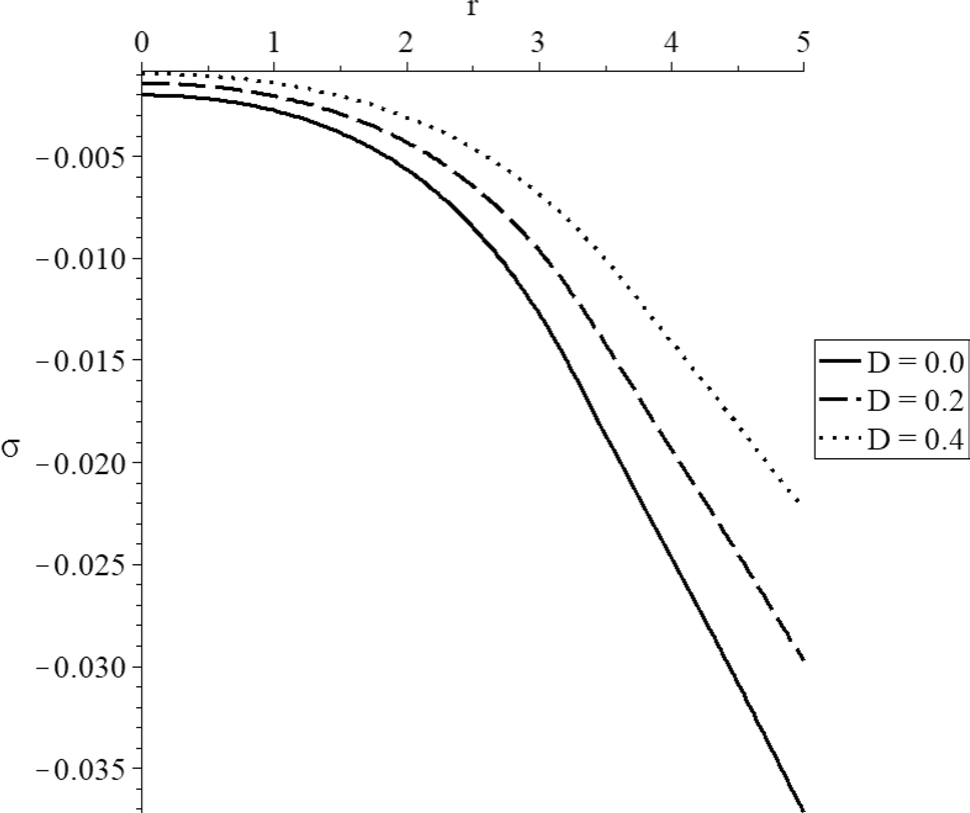
The average stress distribution with various values of the mechanical damage parameter.

Figures 8, 9, 10, 11, 12, 13 have been carried out for various values of the mechanical damage parameter $D = \left( {0.0,0.2,0.4} \right)$ and without rotation $\Omega = 0.0$ in the context of the hyperbolic two-temperature model to stand on its effects on all the studied functions. The case $D = 0.0$ represents the sphere without mechanical damage, while the cases $D = \left( {0.2,0.4} \right)$ represent the sphere with different values of the mechanical damage parameter.

Figures 9 and 10 show that the mechanical damage parameter has minimal effects on the dynamical and conductive temperature increment. This result was expected because of the effect of the mechanical damage parameter exists in the stress–strain relation.

Figure 11 represents that the mechanical damage parameter has significant effects on the volumetric strain distributions. The three curves start from the position $r = 5.0$ with the zero values. Each curve has one peak point, and the absolute values of the peak point take the following order:

76 $$ \left| {e_{\max } \left( {D = 0.0} \right)} \right| > \left| {e_{\max } \left( {D = 0.2} \right)} \right| > \left| {e_{\max } \left( {D = 0.4} \right)} \right| $$

Figure 12 represents that the mechanical damage parameter has significant impacts on the displacement distributions. The three curves start from the position $r = 5.0$ with the zero value. Each curve has one peak point, and the absolute values of the peak point take the following order:

77 $$ \left| {u_{\max } \left( {D = 0.0} \right)} \right| > \left| {u_{\max } \left( {D = 0.2} \right)} \right| > \left| {u_{\max } \left( {D = 0.4} \right)} \right| $$

Figure 13 represents that the mechanical damage parameter has significant effects on the average stress distributions. The three curves start from the position $r = 5.0$ with different values. The absolute values of the start point of the average stress take the following order:

78 $$ \left| {\sigma_{{{\text{r}}\,{ = }\,{5}}} \left( {D = 0.0} \right)} \right| > \left| {\sigma_{{{\text{r}}\,{ = }\,{5}}} \left( {D = 0.2} \right)} \right| > \left| {\sigma_{{{\text{r}}\,{ = }\,{5}}} \left( {D = 0.4} \right)} \right| $$

**Figure 14:**
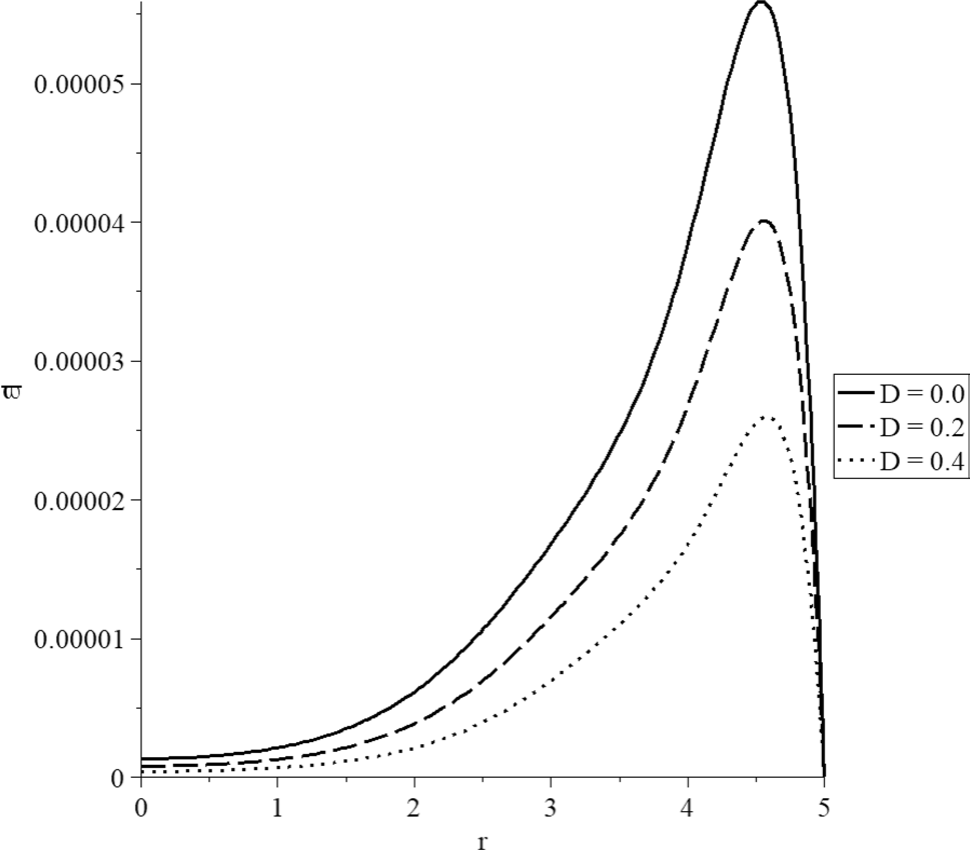
The stress-strain energy distribution with various values of the mechanical damage parameter.

Figure 14 represents that the mechanical damage parameter has significant effects on stress–strain energy distributions. The four curves start from the position $r = 5.0$ with zero values, and each curve has a peak point. The values of peak points of the stress–strain energy take the following order

79 $$ \varpi_{\max } \left( {D = 0.0} \right) > \varpi_{\max } \left( {D = 0.2} \right) > \varpi_{\max } \left( {D = 0.4} \right) $$

**Figure 15:**
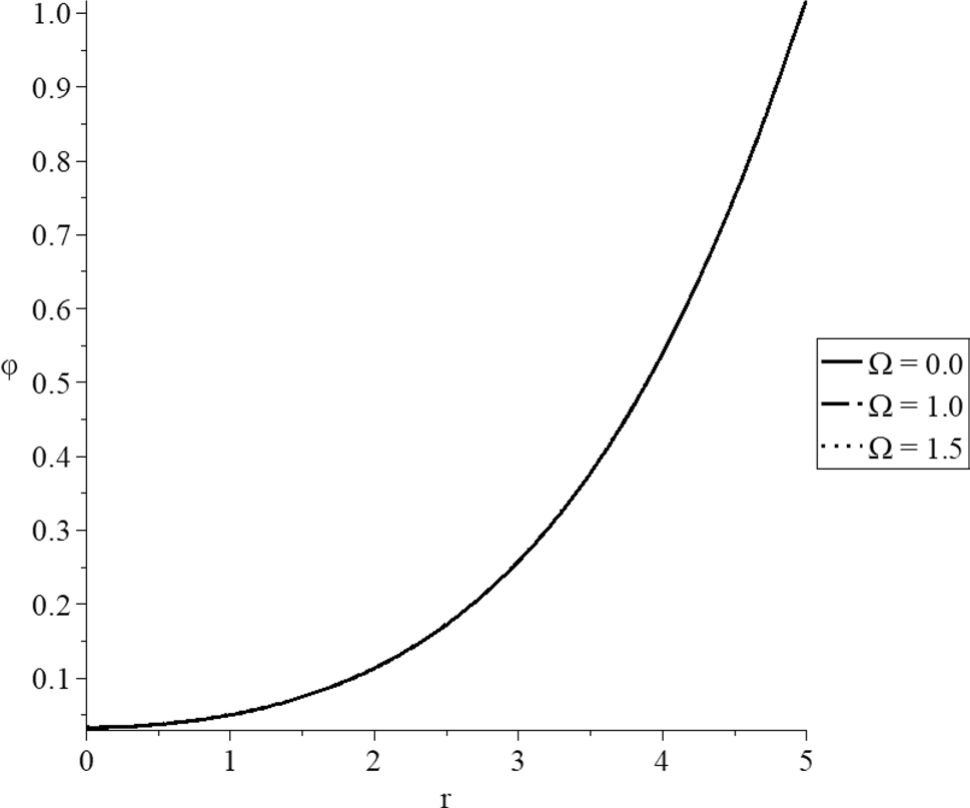
The conductive temperature increment distribution with various values of the angular velocity of the rotation parameter.

**Figure 16:**
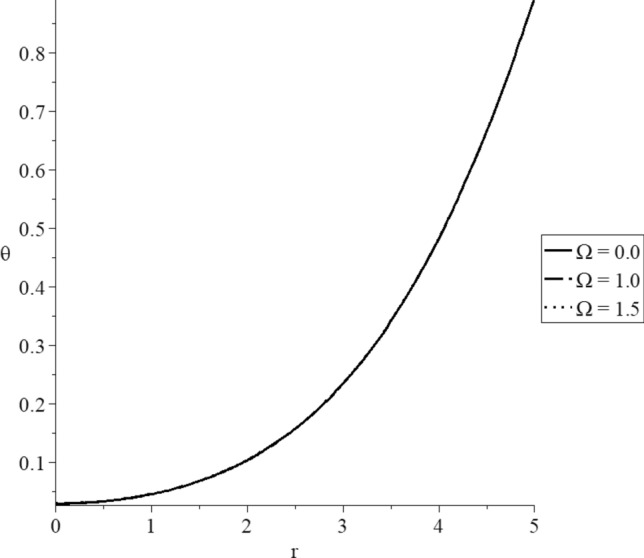
The dynamical temperature increment distribution with various values of the angular velocity of the rotation parameter.

**Figure 17:**
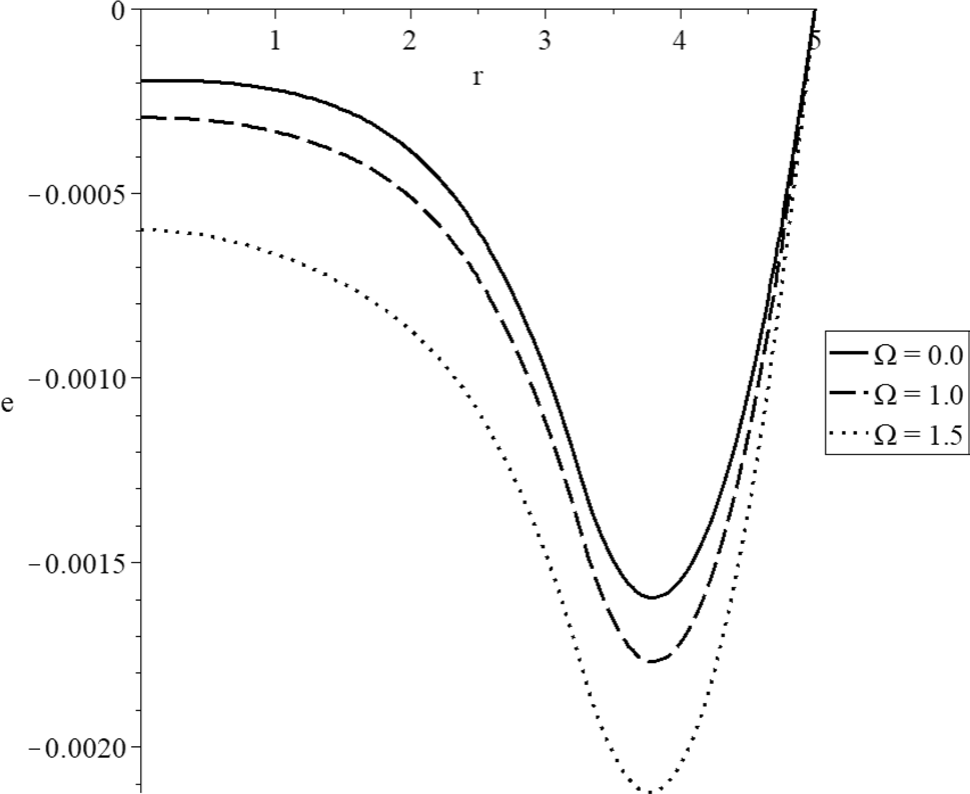
The volumetric deformation distribution with various values of the angular velocity of the rotation parameter.

**Figure 18:**
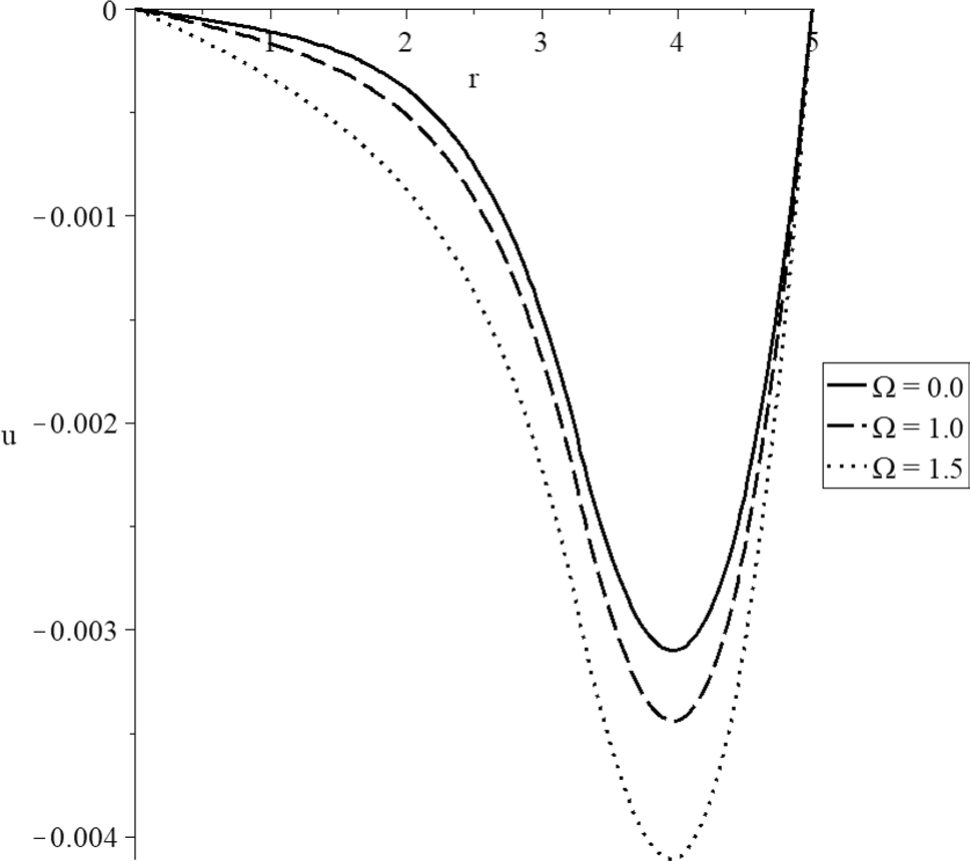
The displacement distribution with various values of the angular velocity of the rotation parameter.

**Figure 19:**
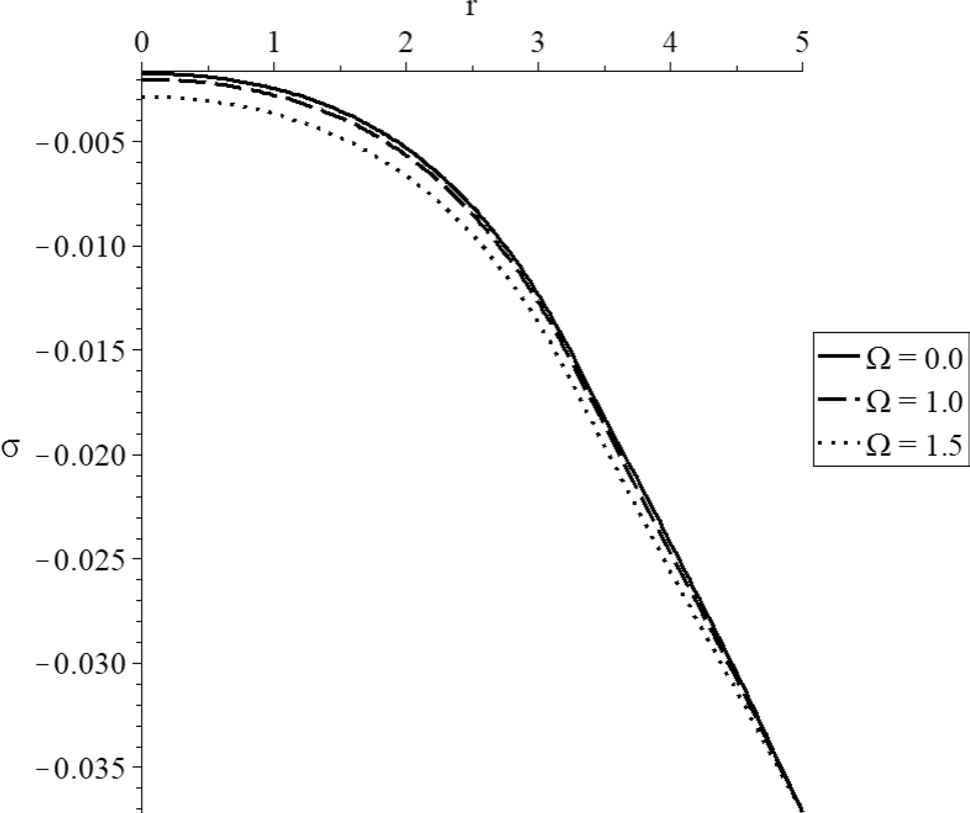
The average stress with various values of the angular velocity of the rotation parameter.

**Figure 20:**
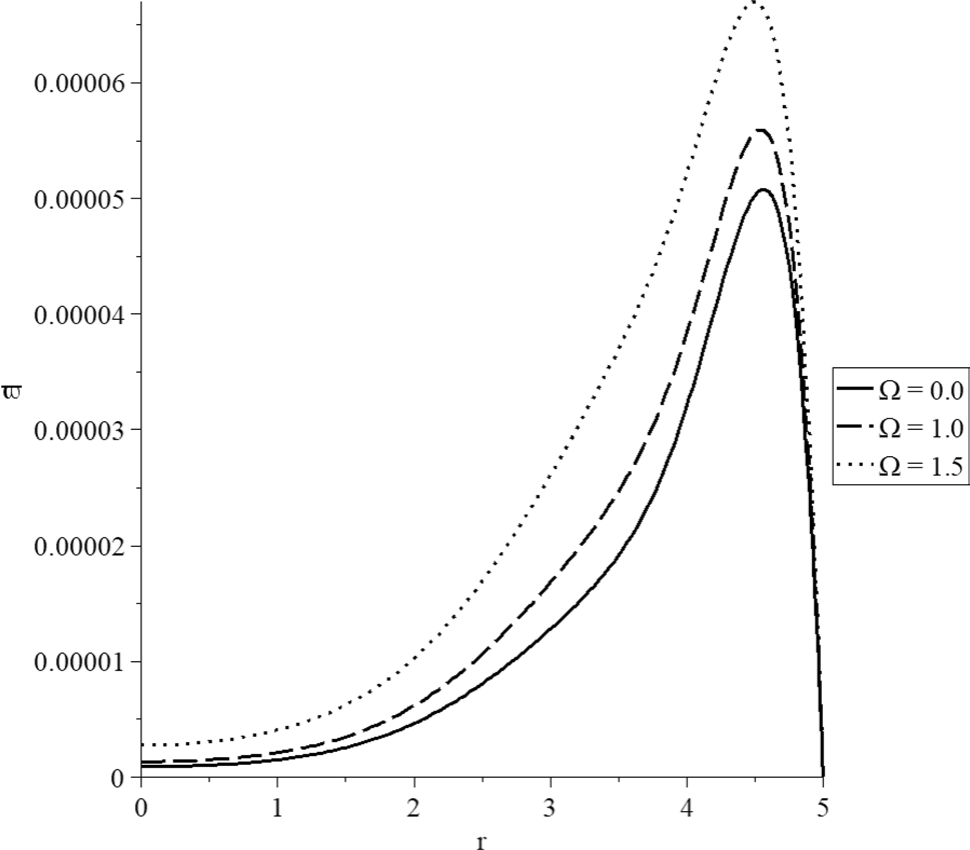
The stress-strain energy distribution with various values of the angular velocity of the rotation parameter.

Figures 15, 16, 17, 18, 19 and 20 have been carried out for various values of the angular velocity parameter $\Omega = \left( {0.0,1.0,1.5} \right)$ and without mechanical damage $D = 0.0$ in the context of the hyperbolic two-temperature model to stand on its effects on all the studied functions. The case $\Omega = 0.0$ represents the sphere without rotation, while the cases $D = \left( {1.0,1.5} \right)$ represent the sphere with different values of the angular velocity parameter.

Figures 15 and 16 show that the angular velocity parameter has minimal effects on the dynamical and conductive temperature increment.

Figure 17 represents that the angular velocity parameter has significant effects on the volumetric strain distributions. The three curves start from the position $r = 5.0$ with zero values, and each curve has a peak point. The absolute value of the deformation increases when the value of the angular velocity parameter increases. The absolute value of the peak points of the volumetric deformation take the following order

80 $$ \left| {e_{\max } \left( {\Omega = 1.5} \right)} \right| > \left| {e_{\max } \left( {\Omega = 1.0} \right)} \right| > \left| {e_{\max } \left( {\Omega = 0.0} \right)} \right| $$

Figure 18 represents that the angular velocity parameter has significant effects on the displacement distributions. The three curves start from the position $r = 5.0$ with zero values, and each curve has a peak point. The absolute value of the displacement increases when the value of the angular velocity parameter increases. The absolute value of the peak points of the displacement takes the following order

81 $$ \left| {u_{\max } \left( {\Omega = 1.5} \right)} \right| > \left| {u_{\max } \left( {\Omega = 1.0} \right)} \right| > \left| {u_{\max } \left( {\Omega = 0.0} \right)} \right| $$

Figure 19 represents that the angular velocity parameter has significant effects on the average stress distributions. The absolute value of the average stress increases when the value of the angular velocity parameter increases.

Figure 20 represents that the angular velocity parameter has significant effects on stress–strain energy distributions. The three curves start from the position $r = 5.0$ with zero values, and each curve has a peak point. The values of the peak points of the stress–strain energy take the following order

82 $$ \varpi_{\max } \left( {\Omega = 1.5} \right) > \varpi_{\max } \left( {\Omega = 1.0} \right) > \varpi_{\max } \left( {\Omega = 0.0} \right) $$

For the validation of the results, one can see that the current results of one-temperature and classical two-temperature agree with the results in references^32–34^.

## Conclusions

The numerical results conclude that the one-temperature model and the hyperbolic two-temperature model of thermoelasticity generate thermal and mechanical waves that propagate with finite speeds. Hence, the hyperbolic two-temperature thermoelasticity model is a successful model to describe thermoelastic materials' thermodynamical behavior.

The hyperbolic two-temperature parameter has significant effects on all the studied functions. The angular velocity parameter and the mechanical damage parameter significantly affect the strain, displacement, stress, and stress–strain energy. In contrast, they have minimal effects on the conductive and dynamical temperature increments.

In the center of the sphere, conductive temperature increments based on one-temperature and hyperbolic two-temperature models disappear before their values from the classical two-temperature model. Therefore, the thermal wave due to the conductive temperature propagates with a finite speed in the form of one-temperature and hyperbolic two-temperature models. In the sense of the classical two-temperature model, instead, it propagates with infinite speed.

The hyperbolic two-temperature thermoelasticity theory introducing a successful model in which the thermoelastic wave propagates with a finite speed.

## Abbreviations

$C_{E} \,$: Specific heat at constant strain
$c_{o} \,\,$: $= \sqrt {\frac{\lambda + 2\,\mu }{\rho }}$Longitudinal wave speed
$D$: The mechanical damage parameter
$e_{ij}$: The strain components
$$K$$: Thermal conductivity
$T_{D} ,\,T_{C} \,\,$: Dynamical and conductive temperature, respectively
$$T_{o}$$: Reference temperature
$$t\,$$: Time
$u_{ij}$: The displacement components
${\upalpha }_{{\text{T}}}$: Coefficient of linear thermal expansion
$$\beta$$: $$= \left( {\frac{\lambda + 2\mu }{\mu }} \right)^{{{\raise0.7ex\hbox{$1$} \!\mathord{\left/ {\vphantom {1 2}}\right.\kern-\nulldelimiterspace} \!\lower0.7ex\hbox{$2$}}}}$$
$${\upgamma }$$: $$\left( {3\lambda + 2\mu } \right)\alpha_{T}$$
$\varepsilon$: $= \frac{\gamma }{{\rho \,C_{E} }}$ The mechanical coupling constant (dimensionless)
$\varepsilon_{1}$: $= \frac{{\gamma T_{o} }}{\mu }$ The thermoelastic coupling constant (dimensionless)
$\eta$: $= \frac{{\rho \,C_{E} }}{K}$ The thermal viscosity
$$\lambda \;,\;\mu \,$$: Lamé’s constants
$\Omega$: The angular velocity of the rotation parameter
$$\rho \,$$: Density
$\sigma_{ij} \,$: Components of the stress tensor
$\tau_{0}$: Thermal relaxation time
